# Perforation of barium sulfate enterography in an infant: A case report

**DOI:** 10.1097/MD.0000000000037926

**Published:** 2024-04-26

**Authors:** Yixing Lu, Lixian Mo, Junhong Chen, Wei Peng

**Affiliations:** a Department of Anesthesiology, Maternal and Child Health Hospital of Guangxi Zhuang Autonomous Region, Nanning, China; b Department of Pediatric Surgery, Maternal and Child Health Hospital of Guangxi Zhuang Autonomous Region, Nanning, China; c Department of Pathology, Maternal and Child Health Hospital of Guangxi Zhuang Autonomous Region, Nanning, China.

**Keywords:** barium peritonitis, case report, gastroenterography, infant, multiple organ failure

## Abstract

**Rationale:**

Barium peritonitis is an inflammatory response that occurs when barium accidentally enters the abdominal cavity during a barium test. In extreme circumstances, it has the potential to harm various organs and even result in death.

**Patient concerns:**

A 3-month-old infant was diagnosed with multiple organ failure after severe barium peritonitis.

**Diagnosis:**

Multiple organ dysfunction is associated with barium peritonitis.

**Interventions:**

The infant underwent surgical intervention and received ventilator support, anti-infection therapy, myocardial nutrition, liver and kidney protection, rehydration, circulation stabilization, and other symptomatic supportive care.

**Outcomes:**

The patient experienced clinical death after treatment and resuscitation was unsuccessful.

**Lessons:**

Barium enema perforation complications are uncommon, but can lead to fatal injuries with a high mortality rate. This case highlights the importance of raising awareness among clinicians about the risks of gastroenterography in infants and children and actively preventing and avoiding similar serious complications. The mortality rate can be reduced by timely multidisciplinary consultation and joint management once a perforation occurs.

## 1. Introduction

Barium sulfate is the safest contrast agent for X-ray radiographic examination of the gastrointestinal tract because of its unique inertness, insolubility, non-toxicity, and ability to absorb X-rays in the gastrointestinal tract for image development. Intestinal perforation during barium enema is a rare but serious and life-threatening complication with an incidence of approximately 0.016% to 0.23%.^[1,2]^ A mixture of barium and feces after perforation can cause severe peritonitis and bacteremia, with a mortality rate of approximately 35% to 50%.^[2,3]^ The probability of death is as high as 75% in elderly individuals with comorbidities and underlying diseases.^[4]^ Most clinical reports are in adults or elderly patients, but this case presents severe barium peritonitis resulting from barium sulfate contrast perforation in an infant.

## 2. Case presentation

The child presented to the pediatrician’s office with a history of recurring bowel problems for >2 months. Lower gastrointestinal imaging was used to identify the cause because the child had no specific medical history. During the imaging procedure, the child experienced crying and restlessness and showed swelling and bruising in the lower abdomen and scrotum, which indicated gastrointestinal perforation. Owing to an emergency, the child was admitted to the hospital. Upon physical examination, the patient’s temperature was 37.1 °C, pulse was 180/min, respiration was 40/min, blood pressure was 92/54 mm Hg, SpO_2_ was 100%, CRT was 5 seconds, and weight was 7.0 kg. The child appeared agitated and restless with pale skin, shortness of breath, and signs of suction and concussion. Coarse respiratory sounds were noted in both lungs; however, no rhonchi was detected. The heart rate was 180/min, with a uniform rhythm and forceful heart sounds, without any pathological murmurs. The lower abdomen had swollen and bruised skin owing to bulging. The scrotum and abdomen also showed signs of swelling and bruising, with purple discoloration. The abdomen exhibited bulging and petechiae on the lower abdominal skin, with abdominal muscle tension, nonpalpable liver and spleen, and tympanic tone on percussion. No intestinal sounds were heard. Petechiae were observed on the penis and scrotum with perineal and perianal flushing. The joints did not show any redness or swelling, and the extremities were cool, with bilateral lower extremity edema (−).

**Figure 1.**
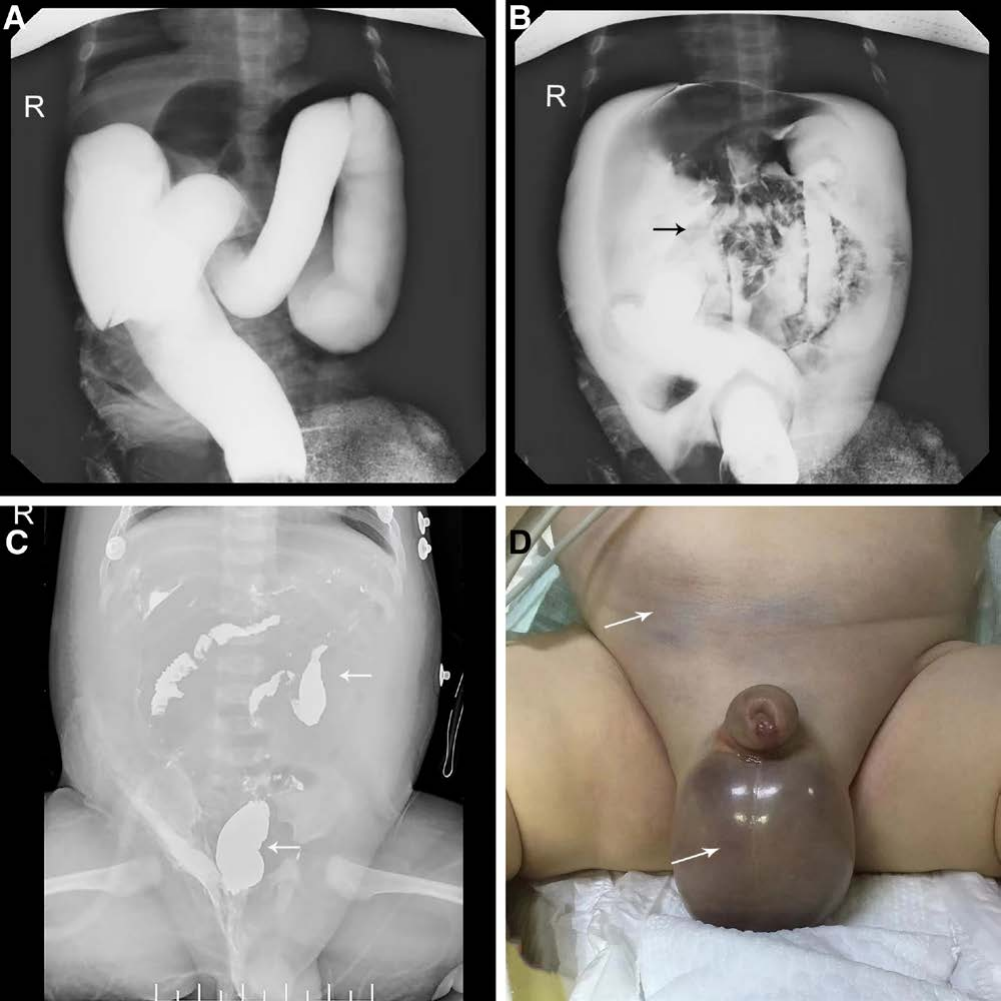
Preoperative abdominal radiographs (A and B). Postoperative abdominal radiographs (C). Preoperative physical manifestations (D).

**Figure 2.**
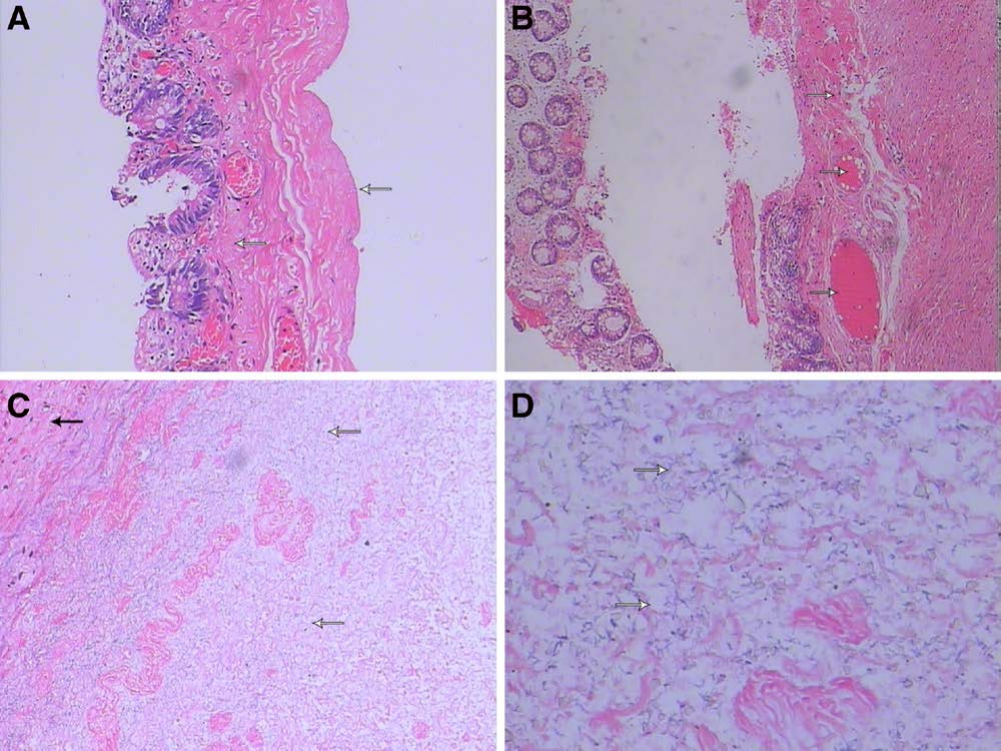
Pathological findings: congestion with hemorrhage in the colon wall, with muscularis propria absent in some areas; fibrous cystic wall-like tissue in the scrotum, with localized coagulation-like necrosis (A: HE ×100; B: HE ×40; C: HE ×100; D: HE ×400).

Lower gastrointestinal barium sulfate contrast radiographs, review of abdominal radiographs on the 2nd postoperative day, preoperative tissue of the lower abdominal wall and scrotum (Fig. 1), and postoperative pathology report (Fig. 2). The patient’s condition progressed rapidly, with multiple organ damage and diffuse intravascular coagulation on the 2nd postoperative day. Complementary check: erythrocytes: 1.8 × 10^12^, hemoglobin: 52 g/L, platelets: 18 × 10^9^, ultrasensitive C-reactive protein: 60.30 mg/L, prothrombin time: 29.4 seconds, PT international normalized ratio: 2.61, activated partial thromboplastin time: 121.5 seconds, fibrinogen: 1.55 g/L, prothrombin time: 79.6 seconds, D-dimer: 8280 ng/mL, aspartate aminotransferase: 1694 U/L, lactate dehydrogenase: 4351 U/L, creatine kinase: 87,898 U/L, creatine kinase isoenzyme: 112 U/L, myoglobin: 1760.86 μg/L, alanine aminotransferase: 610 U/L, creatinine: 101.89 μg/L, uric acid: 1082.37 μmol/L, potassium: 6.9 mmol/L, chloride: 85.5 mmol/L, total calcium: 1.24 mmol/L, and venous blood cultures of *Escherichia coli* and *Enterococcus faecalis*. Electrocardiography suggested wide complex of electrocardiogram tachycardia, elevated T waves, and left deviation of the cardiac axis.

## 3. Treatment

**Figure 3.**
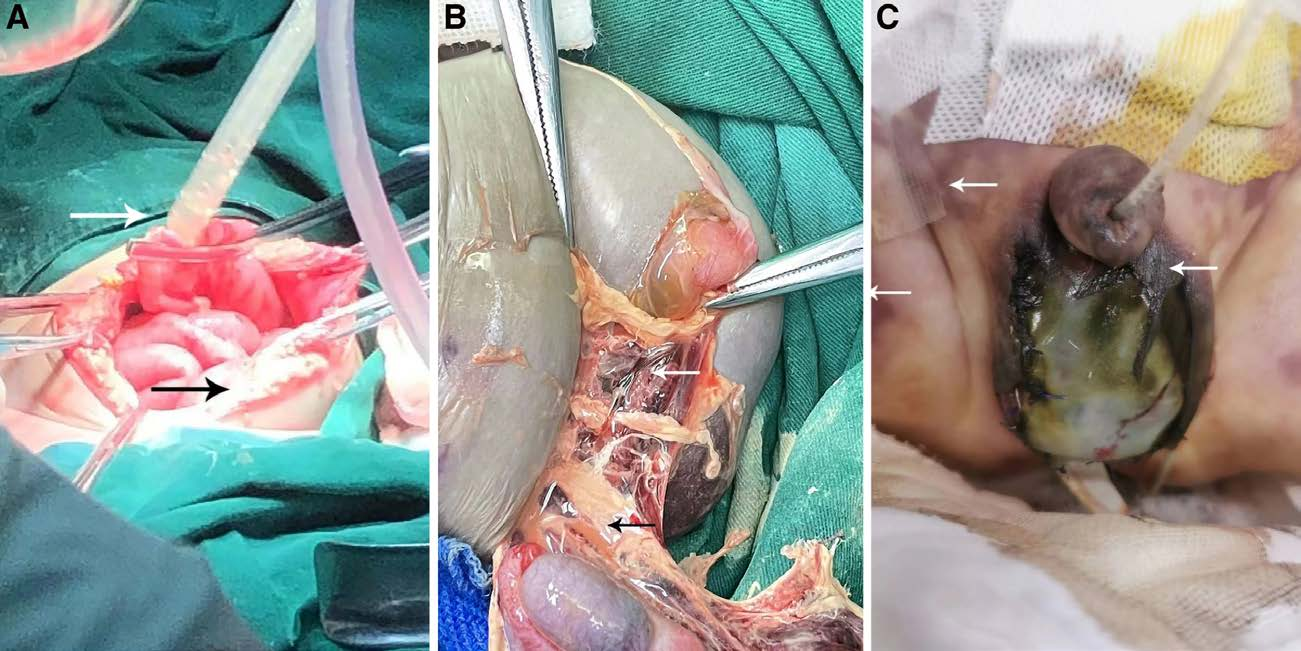
Massive barium leakage from the abdominal cavity and scrotum (A and B) and postoperative scrotal tissue necrosis (C).

Upon admission, the child received ventilator-assisted ventilation (pressure control mode: FiO_2_ 45%, PEEP 4 cm H_2_O, RR 30/min, VT 56 mL), meropenem and vancomycin for antimicrobial therapy, adrenaline and norepinephrine to maintain elevated blood pressure, myocardial nutrition, hepatoprotection, rehydration, and other symptomatic supportive treatments. The child’s condition deteriorated rapidly, and the family consented to an emergency cesarean section, which revealed a significant amount of white barium in the abdominal cavity and a perforation in the middle section of the transverse colon, approximately 2 cm in diameter, with thinning and necrosis of the surrounding intestinal wall. The bilateral scrotal skin appeared whitish and lacked vitality, with edematous endoneurial capsule tissue showing bruise necrosis. The bilateral testicular hematology remained viable, and a substantial amount of barium was observed in the lumen of the right sphincter (Fig. 3). During the operation, the leaked barium was actively removed, and necrotic enterotomy, enterostomy, and bilateral scrotal necrosis dilatation were concurrently performed. Postoperatively, the patient was transferred to the neonatal intensive care unit for further monitoring and treatment. One day after the procedure, the child manifested varying degrees of ischemic necrosis in the penile and scrotal tissues (Fig. 3).

## 4. Discussion

Effective preventive strategies for complications in enema imaging involve lower gastrointestinal angiography with contrast medium infusion through an anal tube with artificially applied perfusion pressure to fill the intestinal lumen. Periodic X-ray imaging is used for intermittent observation of the intestinal tube during contrast enema, as continuous imaging is not used. Complications, such as intestinal perforation, often have delayed detection, at which point a large amount of contrast medium has already leaked outside the intestinal tube. Dynamic subtraction angiography (DSA) is commonly used for systemic vascular examination because of its ability to dynamically display lesion hemodynamics.^[5]^ During contrast enema, DSA allows for dynamic continuous imaging, enabling the timely cessation of contrast agent perfusion and examination termination in the event of intestinal perforation. This strategy effectively prevents excessive leakage of contrast agents and alleviates a mixture of fecal matter and contrast peritonitis. Further clinical research is needed to better understand the causes of peritoneal peritonitis and confirm the effectiveness and safety of this technology. Zhang et al^[6]^ conducted a retrospective analysis of ultrasound images of children undergoing ultrasound-guided saline enemas for the treatment of ileocecal intussusception. This study discovered that ultrasound can effectively predict the outcome of enemas. Diagnostic ultrasound enables real-time dynamic visualization of the intestinal lumen and can serve as a practical complementary method in enterography.^[7]^ Additionally, animal experiments by Sisel et al^[8]^ revealed that the survival rate of animals with peritonitis caused by barium mixed with feces after intestinal perforation was only 10%, whereas those with peritonitis due to water-soluble media mixed with feces had a survival rate of up to 50%. Hernanz-Schulman et al^[9]^ injected barium sulfate mixed with feces into the peritoneal cavity of guinea pigs and observed a mortality rate as high as 95% within 48 hours, much higher than that of guinea pigs with water-soluble iodinated media. For high-risk groups, such as infants, children, and elderly individuals with poor health, gastrointestinal imaging can be achieved by switching to a water-soluble contrast medium instead of barium sulfate, and the use of diluted water-soluble contrast medium is less harmful than barium in causing contamination of the peritoneal cavity.^[10]^

The clinical presentation of barium enema perforation varies depending on the patient’s age and ability to communicate. Pain is the initial symptom in most patients, whereas infants and young children may show irritability and crying. Tachycardia, fever, bacteremia, and infectious shock can develop hours later.^[11]^ If not effectively managed, extensive bacterial toxin release and absorption, along with abdominal inflammation, can lead to multi-organ damage, such as heart, lung, liver, and kidney failure. The severity is influenced by the rupture site and the composition and volume of the leaked contrast medium. Experimental studies have shown that the combined presence of barium and unsterilized feces in the peritoneal cavity can rapidly cause death, with the associated peritonitis being more severe than that caused by either substance alone.^[12]^

Additionally, perforation of a barium enema in children can exhibit specific indicators such as discoloration of the lower abdominal wall and scrotum, as observed in this case (Fig. 1D). Infants and children with a normal inguinal region may possess a patent processus vaginalis (PPV), with the prevalence of PPV in children <2 years old ranging from 27% to 56% and PPV primarily occurring on the right side.^[13]^ The vaginal protrusions are linked to the abdominal cavity. In cases where barium sulfate enema perforates, a significant amount of barium spills into the abdominal and pelvic cavities. Additionally, the abdominal pressure increases when the child cries, causing barium to enter the scrotum along the vaginal protrusion. During the operation in the present patient, a substantial amount of barium infiltration into the scrotum on the right side was observed, but not on the left side, consistent with the aforementioned study report. Barium sulfate is insoluble in water and can be safely retained in the gastrointestinal tract. While the majority of barium is excreted in feces, a small amount may lead to deposition.^[14]^ However, leakage of barium sulfate into the abdominal cavity or other tissues and organs can result in severe complications. The child in this case presented with persistent discoloration and necrosis in the lower abdomen, scrotum, and penile tissues postoperatively (Fig. 3C), indicating that infiltration or residual barium mixtures can induce a strong toxic reaction in the soft tissue. Grable and Moazam^[15]^ reported a case of a child who underwent surgical treatment for barium peritonitis and postoperatively developed a swollen right scrotum with localized redness and tenderness, similar to the signs observed in this case. Barium sulfate enema perforation in adults has not been reported, as the prevalence of PPV significantly decreases in adulthood, and the abdominal and pelvic cavities of adults are much larger in volume, providing ample space to accommodate any barium leakage.

The management and treatment of barium enema perforation involve categorizing perforations as intraperitoneal or extraperitoneal. Therefore, early diagnosis should be followed by immediate and effective treatment. Retroperitoneal or intramural perforation with barium granuloma encased in a thick fibrous capsule and a mild peripheral inflammatory reaction can be conservatively treated with bowel rest, total parenteral nutrition, intravenous fluid therapy, and broad-spectrum antibiotics. It may also be secondary to the development of chronic abscesses, rectal stenosis, or fistulae, which require further treatment based on disease progression.^[16]^

Numerous animal experiments and clinical reports have demonstrated that intraperitoneal perforation can lead to severe peritonitis with a relatively high morbidity and mortality rate.^[9,12]^ Immediate surgical intervention is essential for patients with intraperitoneal perforation combined with severe peritonitis to remove as much intraperitoneal barium as possible, perform thorough irrigation and drainage of the abdominal cavity, and thoroughly remove necrotic tissue.^[11]^ Incomplete removal of barium from the abdominal cavity may result in a persistent inflammatory response that is unresponsive to antibiotic treatment, and the use of steroids should be considered. Kojima et al^[17]^ recommended the use of methylprednisolone 500 mg/d for 3 days, followed by gradual tapering of the dosage, which has been effective in controlling inflammatory responses caused by residual barium; however, the specific type of steroids and dosage is not yet certain, and it is important to rule out the presence of a potential source of infection.

After the initiation of this case, the infant was actively rehydrated and antibiotics were administered to control the infection, improve circulation, and correct disturbances in the internal environment. An emergency cesarean section was performed, yet the infant’s condition continued to deteriorate, ultimately resulting in death. In this situation, assuming a head-down position after onset may decrease the barium dose entering the scrotum. Severe peritoneal inflammation and organ toxicity damage were evident, indicating rapid disease progression and a critical condition. For patients with substantial barium leakage into the peritoneum, prompt execution of cesarean section is crucial.

## 5. Conclusion

Complications arising from barium enema perforation are infrequent; however, the elevated fatality rate warrants the attention of pediatricians, intensive care specialists, and radiologists. Survivors of intestinal perforation may also develop numerous complications. Therefore, it is imperative to meticulously conduct gastrointestinal imaging, opt for a flexible intestinal tube and appropriate filling pressure, and employ DSA or ultrasound for diagnostic assistance. Special consideration should be given to infants, young children, and the elderly with compromised physical health. The cautionary use of barium sulfate enemas is advised for patients with intestinal obstruction and chronic gastrointestinal inflammation.

## Acknowledgments

We thank the Department of Radiology for the images, the Department of Critical Care Medicine for the case information, and the guardian of the child for uniformly reporting this case.

## Author contributions

**Conceptualization:** Yixing Lu.

**Data curation:** Lixian Mo, Junhong Chen.

**Supervision:** Wei Peng.

**Writing – original draft:** Yixing Lu.

**Writing – review & editing:** Yixing Lu, Wei Peng.
